# Factors Associated With Erectile Dysfunction in Men: A Cross-Sectional Study From Balochistan, Pakistan

**DOI:** 10.7759/cureus.76928

**Published:** 2025-01-04

**Authors:** Noman Sadiq, Jamshed Warsi

**Affiliations:** 1 Department of Physiology, University of Sindh, Jamshoro, PAK

**Keywords:** adult, erectile dysfunction, healthcare facilities, smoking, testosterone

## Abstract

Background: Erectile dysfunction (ED) in men is overlooked and is often linked with psychogenic causes. Due to cultural barriers, this area of research remains neglected.

Objective: The study was conducted to determine the factors that can be associated with ED in otherwise apparently healthy men.

Methods: We conducted our cross-sectional case-control study at the teaching hospital (Mekran Medical College) Turbat for six months from March 2023 to September 2023. After obtaining informed consent, 119 subjects aged less than 48 years were included using a convenient purposive sampling technique. Subjects suffering from any chronic disease like diabetes, hypertension, or chronic renal disease were excluded. The sociodemographic data of the participants were recorded. Patient's blood samples were taken to analyze serum testosterone levels. IBM SPSS Statistics for Windows, Version 26 (Released 2019; IBM Corp., Armonk, New York, United States) was used for the analysis of data. The chi-square test and an independent sample t-test were applied to analyze the data.

Results: Among 119 study participants, 65 participants had ED. A significant association of ED was found with the area of residence (OR: 1.60, 95% CI: 1.04-2.48, p-value: 0.031) and smoking status (OR: 3.68, 95% CI: 1.66 - 8.12, p-value: 0.001). A significant difference in the mean age, duration of marriage, and testosterone level was found between subjects with and without ED (p < 0.05).

Conclusion: Men do suffer from ED. Smoking and low levels of testosterone increase ED in men. Effective healthcare strategies should be implemented to address the issue of ED in men.

## Introduction

Erectile dysfunction (ED) is defined as the inability to obtain or maintain an erection sufficient for satisfactory sexual performance [1]. ED can be labeled as psychogenic, organic (drug-induced, cavernosal, vascular, hormonal, and neurogenic), or mixed (psychogenic and organic). A lack of erections characterizes organic ED [2,3]. Vasculogenic ED is characterized by abnormalities in the input or outflow of the penile arteries or veins, and it accounts for 15-72 percent of these organic ED cases [4].

A growing number of younger men are reporting ED, which could have a detrimental impact on their quality of life, even though ED is more common in older men [5]. A review of the literature has shown that 9.8 percent of young men have ED [6]. Among patients who had recently developed ED, one in every four was less than 40 years old, and nearly 50 percent of these young men reported having experienced severe ED [7]. One in every four men in Italy who seek treatment for ED is under the age of 40. Men younger than 40 have been more likely to seek medical treatment for ED since 2011 [8]. The percentage of young men who visited the clinic to seek treatment for ED increased from five percent to more than 15 percent between the years 2010 and 2015. There is a possibility that the prevalence of ED in the current population could approach 30 percent [9].

An increasing percentage of men in Pakistan are expressing concerns regarding their ability to maintain an erection [10,11]. Over the past few years, there has been a significant increase in the number of men under the age of 40 who have ED [8]. ED was primarily thought to be caused by psychological factors in men under the age of 40, especially in Asian countries. Pakistani researchers Khanzada et al. estimated that 51% of men suffer from nonorganic ED. Additionally, compared to patients older than 40, those younger than 40 were more likely to have psychological problems, accounting for 70% of all cases [11].

The International Index of Erectile Function-5 (IIEF-5) is a short, validated questionnaire designed to assess the severity of ED and its impact on sexual health. It consists of five questions focusing on erectile function, orgasmic function, sexual desire, intercourse satisfaction, and overall satisfaction. Each question is scored on a 5-point Likert scale, with a total score ranging from 5 to 25. Lower scores indicate more severe ED. The IIEF-5 is widely used in clinical practice and research to diagnose ED, monitor treatment progress, and evaluate its prevalence in various populations [5]. Discussing ED is often considered taboo in Asian countries like Pakistan due to cultural, religious, and societal norms that prioritize modesty and discourage open conversations about sexual health. The stigma surrounding sexual topics is further fueled by a lack of education, conservative traditions, and the fear of social judgment or ridicule [10,11].

The quality of life for men is greatly affected by ED. ED has repercussions that reach beyond the health of the individual struggling with it. While ED has a significant monetary burden on the state, its emotional impact on individuals is paramount [12]. Discussing ED is still considered a taboo, especially in Asian countries. Very limited studies have been conducted on this topic in Pakistan to date. The objective of our current study is to determine factors associated with ED among adult men. 

## Materials and methods

Study design and ethical approval

This cross-sectional case-control study was conducted at the teaching hospital (Mekran Medical College) in Turbat for a period of six months from March 2023 to September 2023 after getting approval from the Director of Research and Graduate Studies, University of Sindh via letter no DRGS-2149 & from the Ethical Review Committee of Mekran Medical College via letter no MMC/ERC/02/2023. A sample size of 136 was calculated by considering the prevalence of ED as 9.8% in young adults using OpenEpi Software (OpenEpi: Open Source Epidemiologic Statistics for Public Health, www.OpenEpi.com) [6].

Inclusion and exclusion criteria

Men aged less than 48 years were included in the study. Men suffering from any chronic disease like chronic kidney disease, hypertension, or diabetes were excluded. Men consuming alcohol, any form of narcotics, or recreational drugs like ice or using exogenous testosterone were also excluded from the study.

Assessment of parameters

Sociodemographic data, including age, area of residence, family type, smoking status, and duration of the marriage, of the participants were recorded in a proforma. Participants were instructed to fill out the IIEF-5 questionnaire. The questionnaire comprised five questions. The score of IIEF-5 ranges from 5 to 25. Participants who scored less than 21 or less were considered as suffering from ED. Testosterone levels follow a diurnal rhythm, peaking in the early morning and gradually declining throughout the day. The participants were instructed to come between 8.00 am and 10.00 am for the collection of blood samples. The blood samples were analyzed for serum testosterone levels.

Data analysis

Data were entered and analyzed using IBM SPSS Statistics for Windows, Version 26 (Released 2019; IBM Corp., Armonk, New York, United States). Qualitative variables, including age, area of residence, family type, and smoking status, were measured in terms of frequencies. The association of ED with age, smoking status, and area of residence was measured by applying the chi-square test. The mean difference in age, duration of marriage, and testosterone level between subjects with and without ED was measured by applying an independent Student t-test.

## Results

Initially, 141 subjects were contacted for the study, 17 subjects refused to take part in the study, while five participants refused to go for blood sampling, so the final sample size came out as 119 with a response rate of 84.39%. The mean age of study participants was 35 years.

The sociodemographic characteristics of the study participants are summarized in Table 1. The mean age of participants was 34.97 ± 6.92 years. The majority resided in urban areas (58.8%), while 41.2% were from rural areas. Most participants belonged to combined families (58%), and 40.3% were smokers. Regarding age distribution, 26.9% of participants were under 30 years, 42.9% were between 30 and 39 years, and 30.3% were aged 40-48 years.

**Table 1:** Sociodemographic data of study participants. The sociodemographic data are expressed in terms of %.

Parameter	Value
Age in years (Mean ± SD)	34.97 ± 6.92 (N = 119)
Residence	
Urban	70 (58.8%)
Rural	49 (41.2%)
Family Type	
Nuclear	50 (42%)
Combined	69 (58%)
Smoking Status	
Smoker	48 (40.3%)
Non-smoker	71 (59.7%)
Age Group	
Less than 30 years	32 (26.9%)
30-39 years	51 (42.9%)
40-48 years	36 (30.3%)

Table 2 highlights significant associations between ED and age, area of residence, and smoking status. The age group was significantly associated with ED (p=0.016), with the highest proportion of cases observed in the 30-39 year age group (28.6%). Smoking status was also significantly linked to ED (p=0.001), with smokers having higher odds of ED (OR = 3.68, 95% CI: 1.66-8.12) compared to non-smokers. Additionally, the area of residence showed a significant relationship (p=0.031), as urban residents had a higher prevalence of ED (37%) than rural residents (17.6%).

**Table 2:** Significant association of erectile dysfunction with age, smoking status, and area of residence. *p < 0.05. The chi-square test was applied.

Variable	Erectile Dysfunction (n=65)	No Erectile Dysfunction (n-54)	Chi-square X^2^	OR 95% CI	p-Value
Age Group					
Less than 30 years	11 (9.2%)	21 (17.6%)	8.29		0.016*
30-39 years	34 (28.6%)	17 (14.3%)			
40-48 years	20 (16.8%)	16 (13.4%)			
Smoking Status					
Smoker	35 (29.4%)	13 (10.9%)	10.86	3.68 (1.66 - 8.12)	0.001*
Non-smoker	30 (25.2%)	41 (34.5%)			
Area of Residence					
Urban	44 (37%)	26 (21.8%)	4.65	1.60 (1.04-2.48)	0.031*
Rural	21 (17.6%)	28 (23.5%)			

Further analysis revealed significant differences in age, duration of marriage, and testosterone levels between participants with and without ED as shown in Table 3.

**Table 3:** Significant difference in age, duration of marriage, and testosterone level between subjects with and without erectile dysfunction. *p < 0.05. An independent sample t-test was applied.

Parameter	Erectile Dysfunction (n=65)	No Erectile Dysfunction (n-54)	p-Value
Age (Years)	36.23 ± 6.28	33.46 ± 7.40	0.029*
Duration of Marriage (Years)	10.30 ± 4.36	8.53 ± 4.52	0.017*
Testosterone Level (nmol/L)	7.56 ± 4.21	13.06 ± 3.70	0.000*

Participants with ED were older on average (36.23 ± 6.28 years vs. 33.46 ± 7.40 years, p=0.029) and had been married for a longer duration (10.30 ± 4.36 years vs. 8.53 ± 4.52 years, p=0.017). Additionally, testosterone levels were markedly lower in those with ED (7.56 ± 4.21 nmol/L) compared to those without ED (13.06 ± 3.70 nmol/L, p<0.001).

## Discussion

ED in men is usually overlooked and is often linked with psychogenic causes. We conducted our study to investigate factors associated with ED in men, including age, smoking status, area of residence, marital duration, and testosterone levels. Our findings underscore the multifactorial nature of ED, aligning with previous studies that have identified similar sociodemographic and clinical determinants.

Age was found to be significantly associated with ED, with the highest prevalence observed among participants aged 30-39 years. This finding diverges from the traditional perception that ED predominantly affects older individuals, suggesting that psychosocial stressors, lifestyle factors, and early onset of risk factors such as smoking may contribute to ED in younger adults. Similar patterns have been reported in studies emphasizing the growing burden of ED among middle-aged populations [8,9].

Smoking was strongly associated with ED in our study, with smokers exhibiting over three times the odds of developing ED compared to non-smokers. Smoking is a well-established risk factor for vascular dysfunction and endothelial damage, which can impair penile blood flow. These findings echo those of previous research, reinforcing the importance of smoking cessation as a preventative strategy for ED [13,14].

ED has been significantly associated with the area of residence [15]. In our study, urban residence was significantly associated with a higher prevalence of ED compared to rural residence. This could be attributed to urban lifestyle factors, such as higher stress levels, sedentary behavior, and dietary habits, which are known contributors to metabolic and vascular conditions linked to ED. However, rural populations may face underreporting of ED due to cultural stigma, which warrants further investigation.

Our analysis also revealed significant differences in marital duration and testosterone levels between participants with and without ED. Prolonged marital duration may reflect accumulated relationship stress or evolving dynamics that contribute to sexual dysfunction. This finding of our study is in line with the findings of the studies conducted by Van Vo et al. and Ma et al. who also reported that the duration of marriage is associated with ED [16,17]. Meanwhile, lower testosterone levels in participants with ED are consistent with its role in regulating sexual desire and ED. These findings align with prior studies emphasizing the interplay between hormonal health and sexual dysfunction [18,19].

The clinical implications of these findings are substantial. Screening for ED should incorporate a comprehensive assessment of lifestyle factors and hormonal profile, particularly among younger individuals and smokers. Public health interventions targeting smoking cessation and awareness of ED in urban settings could play a pivotal role in mitigating its prevalence.

Our study's limitations include its cross-sectional design that precludes the establishment of causality between identified factors and ED. The sample size can be increased further. Despite these limitations, our study has addressed the issue of ED in men which is often considered taboo and possible factors associated with ED.

## Conclusions

Men do suffer from ED. Smoking, urban residence, increase in marital duration, and low levels of testosterone increase ED in men. Our findings highlight the need for targeted interventions addressing modifiable risk factors to improve sexual health outcomes and overall quality of life. Healthcare policies and facilities for improving sexual health should be developed and employed.
